# Macrophage biomimetic nanocarriers for anti-inflammation and targeted antiviral treatment in COVID-19

**DOI:** 10.1186/s12951-021-00926-0

**Published:** 2021-06-10

**Authors:** Qingqin Tan, Lingjie He, Xiaojun Meng, Wei Wang, Hudan Pan, Weiguo Yin, Tianchuan Zhu, Xi Huang, Hong Shan

**Affiliations:** 1 Center for Infection and Immunity, Guangdong Provincial Key Laboratory of Biomedical Imaging, The Fifth Affiliated Hospital of Sun Yat-Sen University, Zhuhai, 519000 Guangdong China; 2 Southern Marine Science and Engineering Guangdong Laboratory, Zhuhai, 519000 Guangdong China; 3 Key Laboratory of Tropical Diseases Control, Ministry of Education, Zhongshan School of Medicine, Sun Yat-Sen University, Guangzhou, 510080 Guangdong China; 4 Department of Endocrinology, The Fifth Affiliated Hospital of Sun Yat-Sen University, Zhuhai, 519000 Guangdong China; 5 Dr. Neher’s Biophysics Laboratory for Innovative Drug Discovery, State Key Laboratory of Quality Research in Chinese Medicine, Macau University of Science and Technology, Macao, 999078 China; 6 The Sixth Affiliated Hospital of Guangzhou Medical University, Qingyuan People’s Hospital, Qingyuan, 511518 Guangdong China

**Keywords:** COVID-19, Cytokine storm syndrome, Anti-inflammation, Antiviral treatment, Biomimetic nanocarriers

## Abstract

**Background:**

The worldwide pandemic of COVID-19 remains a serious public health menace as the lack of efficacious treatments. Cytokine storm syndrome (CSS) characterized with elevated inflammation and multi-organs failure is closely correlated with the bad outcome of COVID-19. Hence, inhibit the process of CSS by controlling excessive inflammation is considered one of the most promising ways for COVID-19 treatment.

**Results:**

Here, we developed a biomimetic nanocarrier based drug delivery system against COVID-19 via anti-inflammation and antiviral treatment simultaneously. Firstly, lopinavir (LPV) as model antiviral drug was loaded in the polymeric nanoparticles (PLGA-LPV NPs). Afterwards, macrophage membranes were coated on the PLGA-LPV NPs to constitute drugs loaded macrophage biomimetic nanocarriers (PLGA-LPV@M). In the study, PLGA-LPV@M could neutralize multiple proinflammatory cytokines and effectively suppress the activation of macrophages and neutrophils. Furthermore, the formation of NETs induced by COVID-19 patients serum could be reduced by PLGA-LPV@M as well. In a mouse model of coronavirus infection, PLGA-LPV@M exhibited significant targeted ability to inflammation sites, and superior therapeutic efficacy in inflammation alleviation and tissues viral loads reduction.

**Conclusion:**

Collectively, such macrophage biomimetic nanocarriers based drug delivery system showed favorable anti-inflammation and targeted antiviral effects, which may possess a comprehensive therapeutic value in COVID-19 treatment.

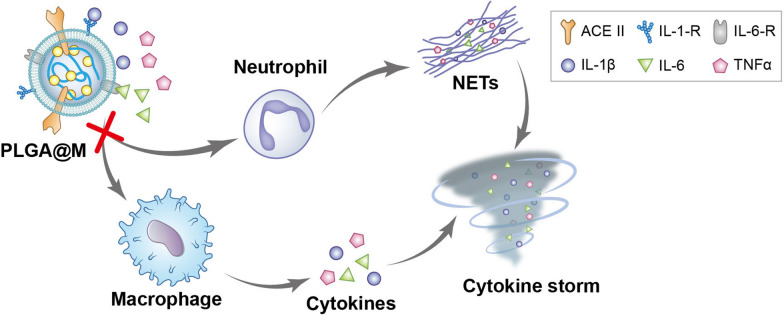

**Supplementary Information:**

The online version contains supplementary material available at 10.1186/s12951-021-00926-0.

## Background

An outbreak of coronavirus disease 2019 (COVID-19) caused by severe acute respiratory syndrome coronavirus 2 (SARS-CoV-2) [1, 2], has provoked a pandemic across the world [3, 4], leading to significant and substantial morbidity and mortality [5–7]. However, thus far, no specific treatment has been proven effective for COVID-19 because of the complex pathogenesis [8–10]. Growing evidence suggest that cytokine storm syndrome (CSS) characterized by excessive inflammation and multi-organs failure is the leading cause of mortality in severe COVID-19 cases [11–16]. Therefore, the strategy to restrain the process of CSS seems a promising way for COVID-19 treatment.

Proinflammatory cytokines IL-6 and IL-1β play a pivotal role in the CSS of COVID-19 [17–19], and the relevant cytokines inhibitors were proposed to relieve symptoms in seriously ill patients [20], such as tocilizumab (a humanized monoclonal antibody against the IL-6 receptor) [21], ruxolitinib (a JAK–STAT inhibitor) and Galectin-3 (both IL-6 and TNF-α inhibitor) [22, 23]. However, efficacy of these inhibitors needs to be determined by further research in clinical practice. Besides, due to the fact that pathological inflammation in COVID-19 is orchestrated by a large number of molecules, inhibiting one or a few cytokines may not suppress inflammation enough to reverse the progression of CSS. Recently, strategies that use the immune cell membranes to coat synthetic nano-cores to manage inflammation have caught much academic attention [24–26]. These nanoparticles inherit the membrane antigenic profile from immune cells, and act as decoys to absorb and neutralize multiple proinflammatory substances from immune cells so as to prevent immune activation [27, 28]. The favorable features of immune cell biomimetic nanoparticles mentioned above inspired us to construct similar systems against the challenge in the treatment of CSS in COVID-19.

Trace to the pathogenesis of severe COVID-19, macrophage seems to be the main immune cell responsible for CSS initiation [29, 30]. When SARS-CoV-2 invade and replicate in host cells, the neighboring macrophages are triggered to generate cytokines and chemokines, leading to accumulation of large amounts of immune cells (including neutrophils and monocytes) in the lung, which ultimately promote further inflammation and result in CSS [31–35]. Besides, the infiltrated neutrophils produce thread-like extracellular structures termed neutrophil extracellular traps (NETs) for virus eradication [36, 37]. However, affected by hyper activated macrophages and persistent infection, excessive NETs are released from neutrophils and induce more inflammation, leading to a further deterioration of CSS [38–40].

**Scheme 1:**
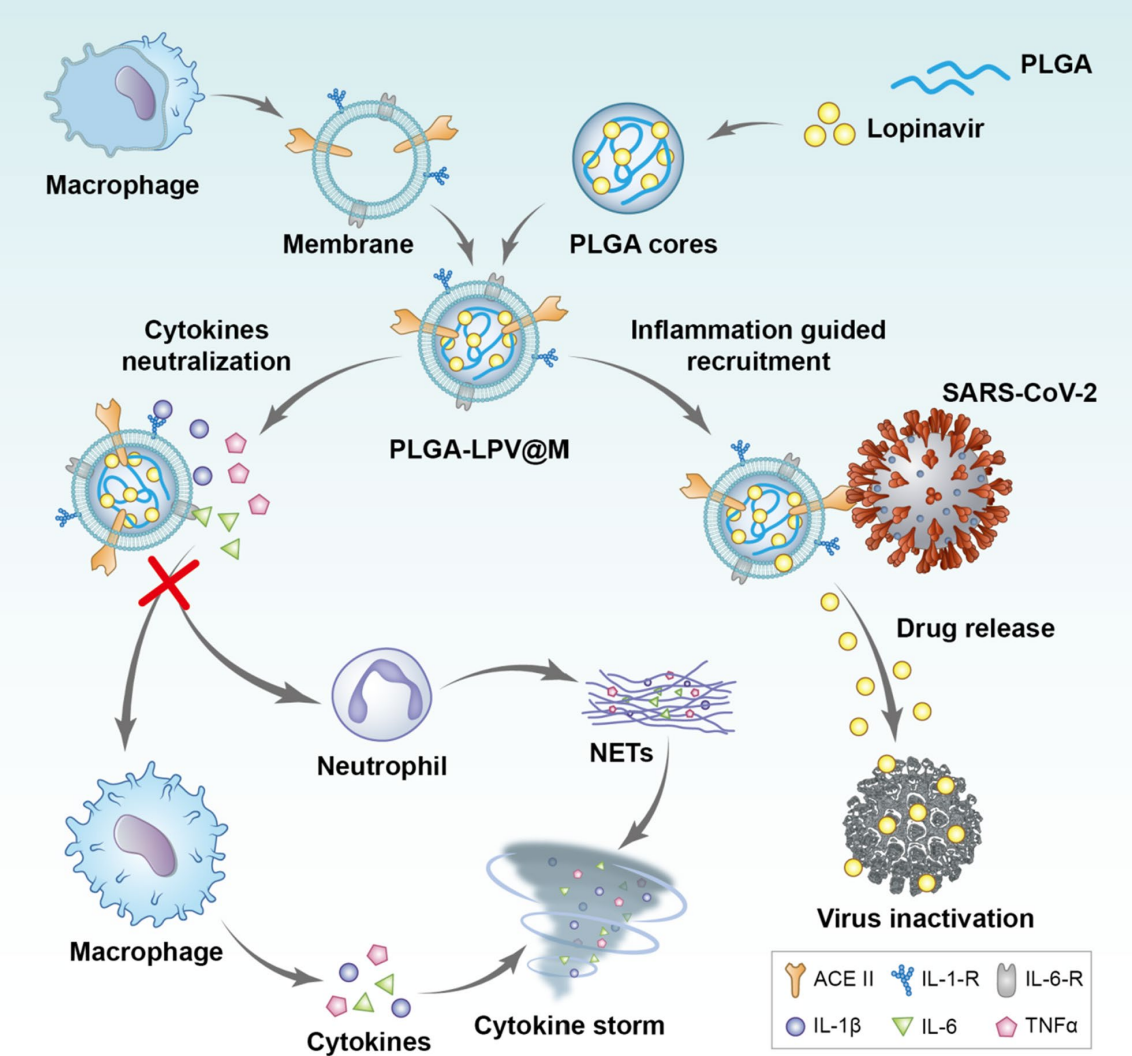
Schematic illustration of macrophage biomimetic nanocarriers based drug delivery system (PLGA-LPV@M) for anti-inflammation and targeted antiviral treatment in COVID-19. PLGA-LPV@M is manifested as a mini macrophage to absorb multiple proinflammatory substances competitively. After that, blocked proinflammatory substances fail to activate macrophages and neutrophils, which reduce the production of cytokines and NETs and alleviate the progression of CSS ultimately. In addition, PLGA-LPV@M could carry drugs homing to the site of viral infection by the inflammatory milieu and EPR effect, and target to the virus, which promote the local accumulation of drugs in the infected tissues, and thus enhance the effectiveness of pharmacotherapy

Given the close correlation of macrophage with the progression of COVID-19, here, we choose macrophage as the membrane donator to establish a macrophage biomimetic nanocarrier based drug delivery system (PLGA@M) for COVID-19 treatment (Scheme 1). In detail, PLGA@M is composed of two parts, one is the macrophage membrane which is used for wrapping on the surface of nanoparticles, and the other is the polymer nanoparticles (PLGA nanoparticles) for drug loading. Due to the surface receptors inherited from macrophage membrane, PLGA@M could disguise itself as a mini macrophage to competitively absorb multiple proinflammatory substances to inhibit the activation of macrophages and neutrophils, and alleviate or prevent the progress of CSS eventually. In addition, driven by the concentration gradient of chemokines and EPR effect (enhanced permeability and retention effect) in the inflammatory environment, PLGA@M could deliver drugs homing into the site of infection. Moreover, the macrophage membranes express the essential SARS-CoV-2 receptor angiotensin-converting enzyme 2 (ACE II), which could target SARS-CoV-2 through the affinity between ACE II and spike protein, so as to improve the efficacy of pharmacotherapy [41–43].

In the study, PLGA@M could suppress the activation of macrophages and neutrophils by neutralizing proinflammatory cytokines IL-6 and IL-1β. Importantly, the formation of NETs induced by COVID-19 patient serum could be reduced by PLGA@M significantly. In addition, lopinavir (LPV), a broad-spectrum antiviral drug which has been proven effective against SARS-CoV-2 in vitro [44, 45], was used as model antiviral drug and loaded in the PLGA@M (PLGA-LPV@M). PLGA-LPV@M exhibited enhanced antiviral efficacy compared with uncoated PLGA-LPV nanoparticles in vitro. In a mouse model of coronavirus infection, compared to the free dye ICG and ICG loaded PLGA nanoparticles (PLGA-ICG NPs) groups, more fluorescence was accumulated in PLGA-ICG@M treated group in the infected organs, suggesting the prominent targeted efficacy of PLGA@M nanocarrier. Moreover, the survival rate of coronavirus infectious mice was improved greatly after PLGA-LPV@M treatment, which might owe to the synergistic effect of anti-inflammation and targeted antiviral treatment. Accordingly, such macrophage biomimetic nanovesicles based drug delivery system may hold great potential in COVID-19 treatment.

## Results and discussion

### Preparation and characterization of PLGA@M and PLGA-LPV@M

**Fig. 1:**
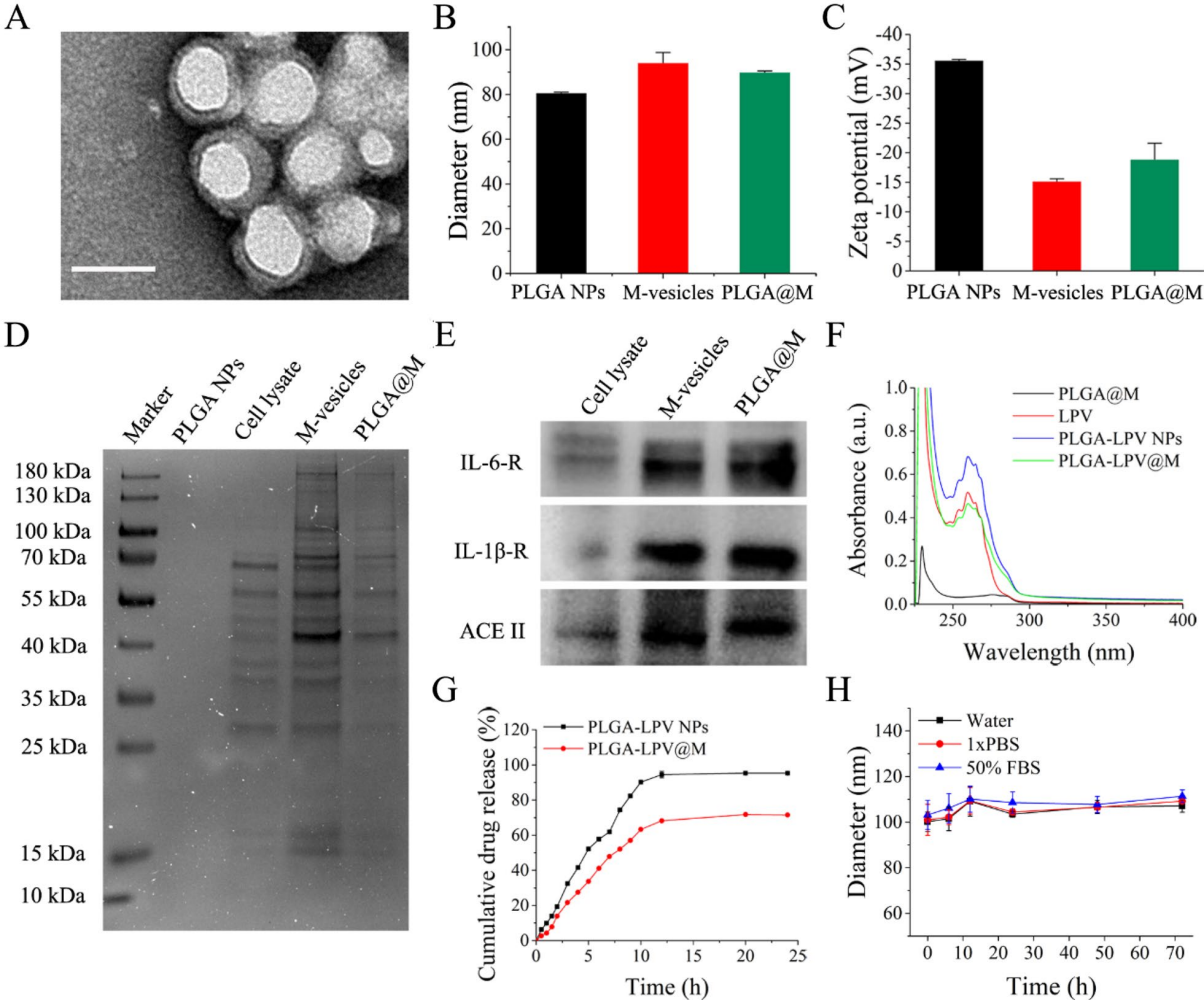
Preparation and characterization of PLGA@M and PLGA-LPV@M. **A** Representative image of PLGA@M examined with transmission electron microscopy. Samples were stained with uranyl acetate. (Scale bar: 100 nm.) **B** Hydrodynamic diameter and **C** zeta potential of PLGA NPs, macrophage membrane vesicles (M-vesicles) and PLGA@M after formulation in water (n = 3). **D** SDS-PAGE electrophoresis protein analysis of cell lysate, M-vesicles and PLGA@M. **E** Western blots analysis for three key surface markers (IL-6-R, IL-1β-R and ACEII) in cell lysate, M-vesicles, and PLGA@M. **F** UV−vis spectra of PLGA@M, lopinavir (LPV), PLGA-LPV NPs and PLGA-LPV@M. **G** Time dependent LPV release profiles from PLGA NPs with (red) or without (black) membranes coating. **H** Stability of PLGA-LPV@M in deionized water, 1× PBS, and 50% FBS determined by monitoring particle size over 3 days

To prepare macrophage biomimetic PLGA@M, membranes derived from human macrophage cell line (THP-1 cells) were fused onto Poly (lactic-*co*-glycolic acid) nanocores (PLGA NPs) by sonication and the optimal PLGA-to-membrane protein weight ratio was explored. As shown in Additional file 1: Fig. S1, there was no obvious difference in diameters of PLGA@M produced at PLGA-to-membrane protein weight ratio of 1:0.5 in both water and 1× PBS, which implied superior colloidal stability under this ratio and was used for further experiments. For the morphology investigation, PLGA@M were stained with uranyl acetate and visualized by transmission electron microscopy (TEM), and the obtained image showed that PLGA@M were spherical in shape around with a monolayer of membranes, which exhibited a typical core–shell structure (Fig. 1A). Meanwhile, as shown in Fig. 1B, after membranes coated, the hydrodynamic sizes of nanoparticles increased from 85.8 ± 4.4 nm to 102.2 ± 4.0 nm, and the surface zeta potential of nanoparticles negative decreased from − 42.4 ± 1.7 mV to − 12.4 ± 1.0 mV (Fig. 1C). These changes were consistent with the addition of a bilayer cell membrane.

To confirm the presence of macrophage membranes on the PLGA@M, protein electrophoresis was conducted to study the protein profile in cell lysate, macrophage membrane vesicles and PLGA@M separately. As shown in Fig. 1D, PLGA NPs had no protein expression because there were no covering membranes. Compared to the cell lysate, macrophage membrane vesicles and PLGA@M were enriched with comparable protein bands, which may be related to the fact that cell lysates contain not only membrane proteins but also abundant cell contents. These results suggested that PLGA@M successfully inherited the membranes from macrophages. Furthermore, western blot verified that CSS related key cytokines receptors including IL-6 receptor (IL-6R) and IL-1 receptor (IL-1R), and SAS-CoV-2 crucial receptor ACE II were expressed on the PLGA@M (Fig. 1E), which implied the potential application of PLGA@M in COVID-19 inhibition treatment.

After confirming the successful fabrication of PLGA@M, LPV loaded macrophage biomimetic nanocarriers (PLGA-LPV@M) were synthesized and characterized. Diameters of PLGA-LPV NPs and PLGA-LPV@M increased slightly compared with the naked PLGA NPs and PLGA@M (Additional file 1: Fig. S2). The loading of LPV was verified by UV–Vis absorption spectroscopy. As shown in Fig. 1F, the absorption peaks of PLGA-LPV NPs and PLGA-LPV@M were consistent with LPV, indicating LPV were successfully encapsulated into the PLGA NPs and PLGA@M. Furthermore, the release profiles of LPV from PLGA NPs and PLGA@M were recorded using a UV–Vis spectrophotometer at the wavelength of 267 nm. The release rate of LPV from PLGA@M was lower than membranes uncoated PLGA NPs. Meanwhile, the cumulative release of LPV from PLGA@M was 71.5% less than 95.3% of PLGA NPs in 24 h (Fig. 1G), suggesting a more prolonged drug release from PLGA@M owing to the coated membranes. Importantly, PLGA-LPV@M exhibited appreciable stability of nanoparticle size over 72 h when suspended in water, 1× PBS and 50% serum, respectively (Fig. 1H).

### Neutralizing proinflammatory cytokines by PLGA@M

**Fig. 2:**
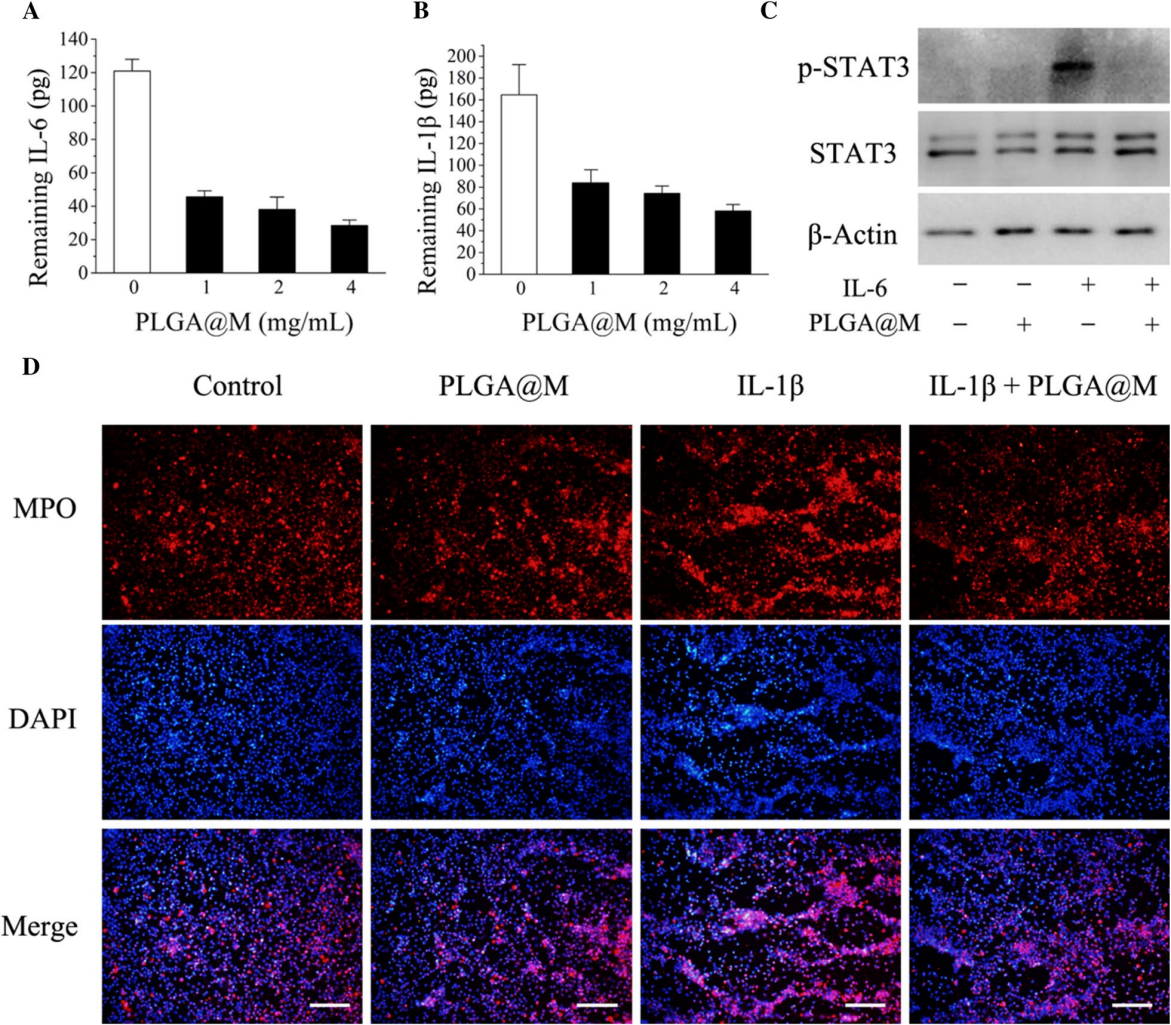
PLGA@M removed the proinflammatory cytokines in vitro. The binding capacity of PLGA@M to recombinant human **A** IL-6 and **B** IL-1β (n = 3). **C** The expression of p-STAT3 protein in macrophages with different treatment was detected by western blot. **D** Fluorescent images of MPO expression (red) in neutrophils after incubation with IL-1β and PLGA@M. (Scale bar: 50 μm.)

Removing overproduced proinflammatory cytokines in the body is essential for CSS alleviation [46]. Here, we assessed the cytokines neutralizing capability of PLGA@M by introducing IL-6 and IL-1β, which were the crucial representative cytokines of CSS in COVID-19. Briefly, human recombinant cytokines (IL-6 and IL-1β) were incubated with different doses of PLGA@M, and the residue of cytokines in the supernatant solution was quantified by enzyme-linked immunosorbent assay (ELISA). As shown in Fig. 2A, B, 4 mg of PLGA@M could remove 93 pg of IL-6 and 107 pg of IL-1β, corresponding to cytokines removal yields of 76.9% and 64.8% respectively, which indicated that PLGA@M could sequester these two cytokines effectively. Next, we further evaluated the capability of PLGA@M to inhibit the activation of macrophage and neutrophil induced by cytokines. STAT3, a member of signal transducers and activators of transcription (STATs) family, has a close relationship with macrophage activation and inflammation development [47, 48]. Here, we detected the phosphorylation levels of STAT3 (p-STAT3) to determine the activation of macrophages. As shown in Fig. 2C, p-STAT3 in macrophages was up-regulated obviously after incubated with IL-6 for 2 h. However, IL-6 induced p-STAT3 in macrophages could be reduced when pretreated with PLGA@M. In addition, myeloperoxidase (MPO) as the biomarker of neutrophils activation was detected in neutrophils under immunofluorescence microscopy [49, 50]. As shown in Fig. 2D, an increased level of MPO (red fluorescence) was expressed in neutrophils after cultured with IL-1β for 4 h, however the elevated expression of MPO induced by IL-1β could be reduced by PLGA@M (Additional file 1: Fig. S3). Therefore, these results indicated that PLGA@M could suppress the cytokines induced activation of macrophages and neutrophils effectively.

### Inhibiting proinflammatory factors of COVID-19 in vitro

**Fig. 3:**
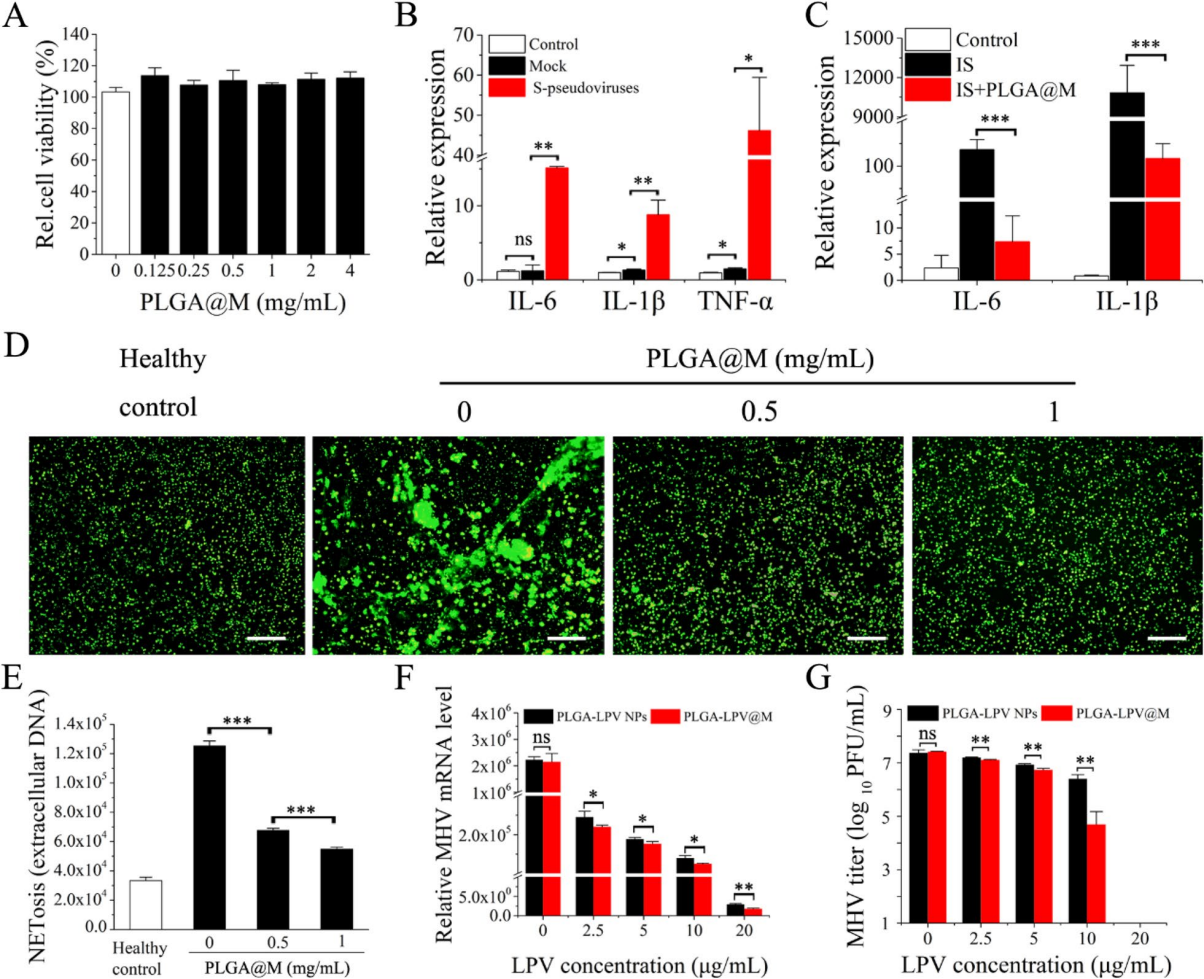
The efficacy of anti-inflammatory and antiviral by PLGA@M in vitro. **A** Cell viability of THP-1 cells after incubated with PLGA@M in different concentrations (n = 6). **B** The mRNA expression level of main proinflammatory cytokines in A549 cells after infected with SARS-CoV-2 pseudoviruses (S-pseudoviruses) (n = 3). **C** The mRNA expression level of IL-6 and IL-1β in macrophages after stimulated with the supernatant of S-pseudoviruses infected A549 cell (IS) with or without PLGA@M treatment (n = 3). **D** Representative images of NETs released from neutrophils induced by COVID-19 patient serum after treated with PLGA@M in different concentrations. NETs were stained green. (Scale bar: 50 μm.) **E** NETosis in neutrophils induced by COVID-19 patient serums was quantified using DNA dye Pico Green after treatment with PLGA@M in different concentrations (n = 3). **F** The virus mRNA level in MHV infected L929 cell after treated with PLGA-LPV NPs and PLGA-LPV@M at different LPV concentration (n = 3). **G** Virus titers in MHV infected L929 cell was determined by the plaque assay after treated with PLGA-LPV NPs and PLGA-LPV@M at different LPV concentrations (n = 3). Data presented as mean ± s.d. *p ≤ 0.05, **p ≤ 0.01, ***p ≤ 0.001. *ns* not significant

After confirming that PLGA@M had no detectable cytotoxicity to human and murine immune cells (Fig. 3A, Additional file 1: Fig. S4), PLGA@M was further explored the effect in the virus infected cell-mediated immune response. As previously reported [30], human lung epithelial cells were activated and released proinflammatory substance into the extracellular environment when undergone SARS-CoV-2 infection, as a result, the extracellular proinflammatory factors recruited and activated macrophages for virus eradiation. However, in severe COVID-19 cases, the activated macrophages may cause pathology in lungs. Hence, it would be advantageous for COVID-19 treatment if the process of virus induced macrophage activation could be inhibited by PLGA@M. Biosafety pseudovirus with SARS-CoV-2 spike protein shared same infection mechanism as SARS-CoV-2, hence, the pseudovirus has been developed as a common alternative model of live SARS-CoV-2 [51]. For instance, Ou et al. identified the SAS-CoV-2 receptor, entry pathway and potential drug targets by using SARS-CoV-2 S protein pseudovirus system [52]. Bayati et al. developed SARS-CoV-2 S protein pseudovirus to study the virus entry, which confirmed that SARS-CoV-2 used clathrin-mediated endocytosis to get into cells [53]. Besides, Wibmer et al. employed pseudovirus model to evaluate the responses of therapeutically relevant antibodies to SARS-CoV-2 [54]. Based on it, pseudoviruses incorporated with SARS-CoV-2 spike protein (S-pseudoviruses) were used to establish the virus infected lung cellular model (A549 cell) to simulate the SARS-CoV-2 infections in human lung epithelial cells. Compared to the mock group which was bald particles with no spike proteins, the significantly elevated expression level of proinflammatory cytokines (IL-6, IL-1β and TNF-α) was observed in S-pseudoviruses infected A549 cells (Fig. 3B), which indicated that the immune response of S-pseudoviruses infected lung cellular model was mainly induced by spike protein rather than lentivirus. After that, we collected the inflammatory supernatant of S-pseudoviruses infected A549 cell to act on macrophages directly, to simulate the activation process of macrophages in patients. As shown in Fig. 3C, the infected supernatant (IS) significantly up-regulated expression of IL-6 and IL-1β in macrophages. While in the presence of PLGA@M, a 100-fold decreased for IL-6 and tenfold decreased for IL-1β expression were detected in macrophages, implying the great capability of PLGA@M in inhibiting the inflammation induced by virus infected cells.

Next, anti-inflammatory effect of PLGA@M was further tested with serum samples which obtained from COVID-19 clinical patients. Patient serum is teeming with inflammatory mediators and cytokines, which could promote neutrophils activation and present a milieu favoring NETosis [40]. Indeed, under NETosis of neutrophils, NETs were released to form inflammation and microvascular thrombosis, which could exacerbate the CSS in COVID-19 patients [55, 56]. Therefore, it would be an inspirational way for COVID-19 treatment if patient serums induced NETs could be suppressed. As shown in Fig. 3E, after 4 h incubation with patient serum, neutrophils from healthy donors were robustly triggered to undergo NETosis, with fivefold externalization of DNA increased. In contrast, when the patient serum was pre-treated with PLGA@M at different concentration for 30 min, they showed decreased externalization of DNA with a dose-dependent inhibition effect. Immunofluorescence microscopy demonstrated similar results that extracellular chromatin structures of NETs were reduced by PLGA@M in a dose-dependent manner (Fig. 3D). Collectively, PLGA@M could suppress the formation of NETs induced by COVID-19 patient serum, which would benefit for anti-inflammation therapy in COVID-19.

### The antiviral activity of PLGA-LPV@M in vitro

Considering that studies with infectious SARS-CoV-2 require rigorous biosafety, mouse hepatitis virus (MHV) also known as mouse coronavirus (MCoV), which is highly homologous to SARS-CoV-2, has brought about widespread attention [57, 58]. Liu et al. suggested that MHV could be used as a valuable tool for the rapid drug screening against SARS-CoV-2, and then the selected drug was further evaluated in SARS-CoV-2 [59]. Besides, MHV was introduced as a suitable surrogate to validate the inactivation of SARS-CoV-2 by UV-C treatment in Pendyala’s work [60]. Thus, we used MHV as a surrogated virus of SARS-CoV-2 to verify the antiviral activity of PLGA-LPV@M. To corresponding to the model mouse virus, we selected murine derived macrophages (RAW264.7 cells) as the membranes source of PLGA-LPV@M. Similarly, 4 mg/mL murine macrophage derived PLGA@M exhibited a cytokine removal yield of 98% for mouse IL-6 and 63.8% for mouse IL-1β (Additional file 1: Fig. S5), which meant that PLGA@M possessed a capacity to neutralize the mouse proinflammatory cytokines as well. Next, MHV was first incubated with PLGA-LPV@M in different dose individually for 1 h, and then the mixtures were added into L929 cells culture plates for 24 h incubation. Considering LPV is a lipophilic drug, free LPV is unstable in aqueous solution, here, water-soluble PLGA-LPV NPs as control group were introduced in this study. As shown in Fig. 3F, both PLGA-LPV NPs and PLGA-LPV@M were able to prevent viral replication in a dose dependent manner, among which, the viral load in PLGA-LPV@M treated group showed slightly lower than PLGA-LPV NPs group. A plausible reason was that PLGA-LPV@M might possess a better affinity to the virus due to the macrophage membranes. Meanwhile, the plaque reduction assay illustrated similar antiviral activity (Fig. 3G).

### Biocompatibility and biodistribution of PLGA@M in vivo

**Fig. 4:**
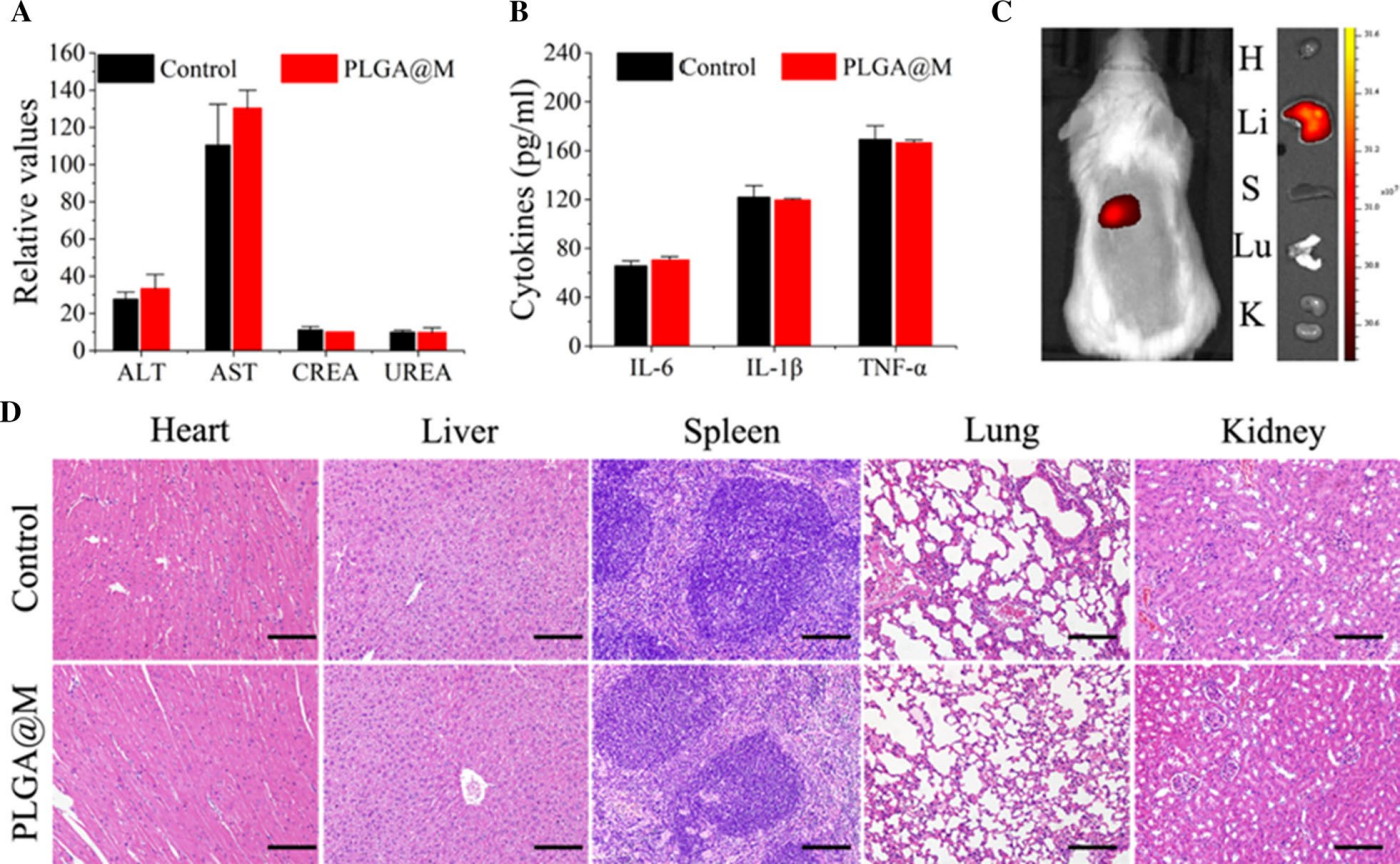
Biocompatibility and biodistribution of PLGA@M in vivo. **A** Blood biochemistry analysis of the healthy BALB/c mice at 21 days after intravenous injected with PLGA@M (n = 3). **B** The serum levels of IL-6, IL-1β and TNF-α in the healthy BALB/c mice after 21 days of PLGA@M treatment (n = 3). **C** The distribution of PLGA-ICG@M in healthy mice by living fluorescence imaging (H: heart, Li: liver, S: Spleen, Lu: lung, K: kidney). **D** Histological images of heart, liver, spleen, lung, and kidney samples from sacrificed mice at 21 days with or without PLGA@M treatment. (Scale bar: 50 μm.)

To systematically estimate the biocompatibility of PLGA@M in vivo, PLGA@M (1 mg/mL, 200 μL) and saline (as control group) were i.v. injected into healthy BALB/c mice individually. Both in control and PLGA@M treated group, the detected values of liver function indices (alanine aminotransferase (ALT), aspartate aminotransferase (AST)), renal function indices (creatinine (CRE) and blood urea nitrogen (BUN)) were all in the permit range (Fig. 4A). Besides, the serum inflammatory cytokines (IL-6, IL-1β and TNF-α) of mice treated with PLGA@M were comparable to those of saline-treated group (Fig. 4B), implying the favorable concealment of PLGA@M in mice immunity system. Furthermore, there was no obvious damage in the main organs (heart, liver, spleen, lung, and kidney) of mice by histological investigation (Fig. 4D). Overall, this set of data supported the good biocompatibility of PLGA@M in vivo.

To study the biodistribution of PLGA@M in healthy mice, PLGA@M loaded with fluorescent dye ICG were synthesized (ICG-PLGA@M). After administration with ICG-PLGA@M 24 h, treated mice were tested by live fluorescence imaging, and then the main organs of mice were harvested. Live images revealed that the fluorescence was mainly accumulated in liver, which implied that liver was the main organ responsible for PLGA@M metabolism (Fig. 4C).

### Targeted delivery in the coronavirus infected mouse model

**Fig. 5:**
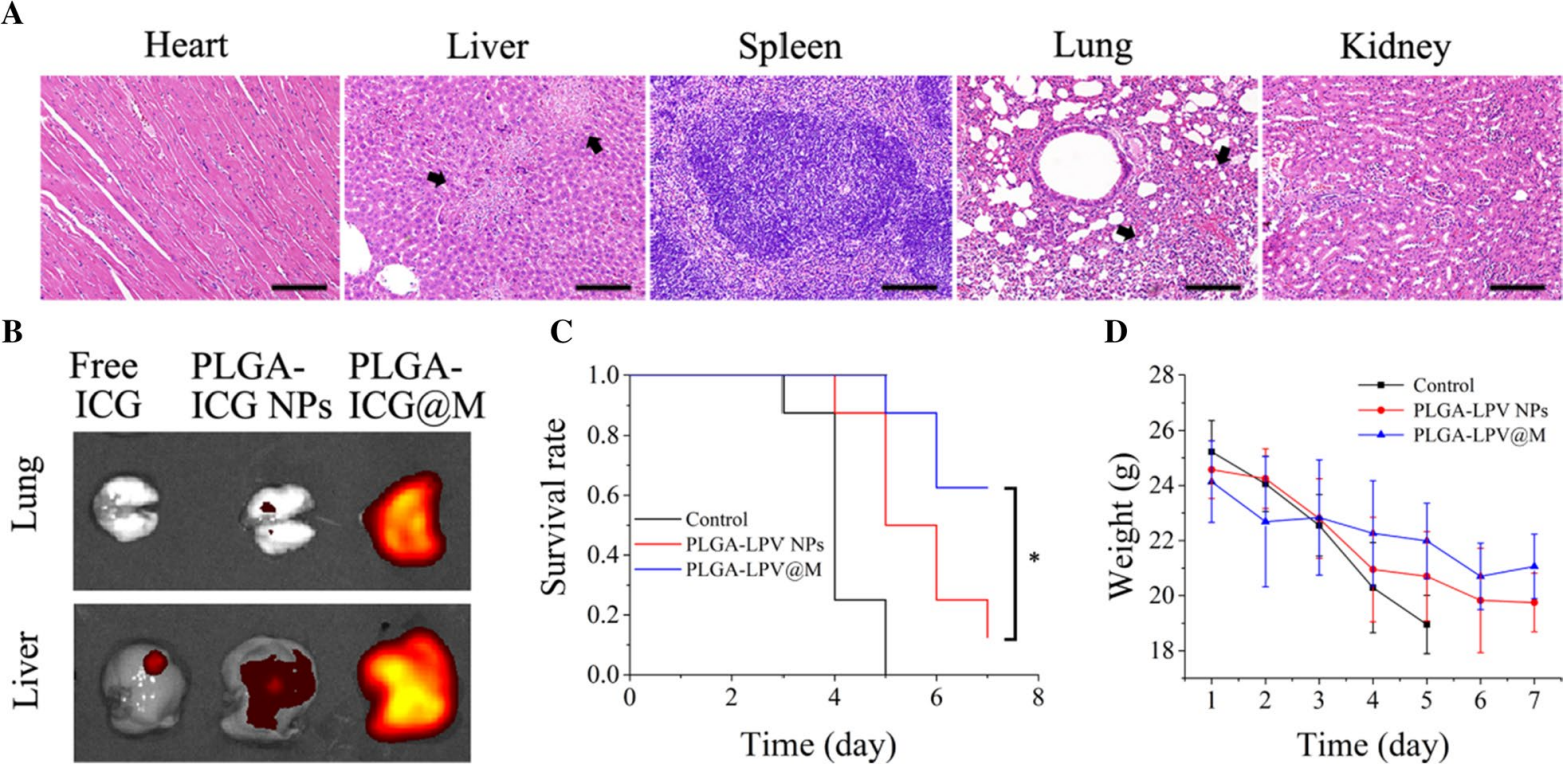
Targeted and therapeutic efficiency of PLGA@M in vivo. **A** Representative histological images of main organs derived from coronavirus infectious mouse model. (Scale bar: 50 μm. Black arrows represent inflammatory injury.) **B** Ex vivo fluorescence bio-imaging analysis of ICG fluorescent signal in livers and lungs of coronavirus infected mouse. **C** Survival curve of severe coronavirus infectious mice after treated with PLGA-LPV NPs, PLGA-LPV@M (n = 8). **D** The weight variations of coronavirus infectious mice after treated with PLGA-LPV NPs and PLGA-LPV@M (n = 8). Data presented as mean ± s.d. *p ≤ 0.05, **p ≤ 0.01, ***p ≤ 0.001

Generally, inflammatory sites enriched with cytokines and chemokines, which generated concentration gradient in blood vessels, leading to the recruitment of macrophages [30]. Hence, we conjectured that PLGA@M disguised as mini macrophage remained the analogous effects, which could carry drugs homing into the inflammation sites as well. MHV was highly susceptible to almost all of mouse strains but safe to human, and MHV induced mouse infections was widely used as a surrogated model for human coronavirus study [61]. As Yang et al. reported MHV respiratory infectious mouse model exhibited acute pneumonia syndrome including respiratory symptoms, elevated cytokines and severe lung injuries, which could closely mimick ARDS caused by SARS-CoV and MERS-CoV infections [62]. Furthermore, Guo’s group established an ARDS mouse model by MHV and SARS-CoV-2, which showed that MHV and SARS-CoV-2 shared an almost uniform immune response [63]. Given the drawback of level three biological confinements and SARS-CoV-2 animal model was difficult to obtain, we constructed the intranasal MHV infected mouse model as SARS-CoV-2 substitution model. As shown in Fig. 5A, coronavirus infected mice had severe inflammatory injury in lung and liver, which was consistent with the pathological features of COVID-19 to a certain extent. Thus, MHV inoculated coronavirus infected mouse model was employed to investigate the targeted delivery efficacy of PLGA@M. In the study, coronavirus infected mice were i.v. injected with free ICG, PLGA-ICG NPs, and PLGA-ICG@M respectively. After 24 h injections, lungs and livers were harvested from all treated mice groups to detect the fluorescence distribution by IVIS system. PLGA-ICG@M treated group exhibited strong fluorescence both in lungs and livers, whereas macrophage membranes uncoated PLGA-ICG NPs treated group showed less fluorescence signals reservation, and few fluorescence was presented in free ICG treated group (Fig. 5B). Moreover, PLGA-ICG@M only mainly accumulated in livers in healthy mice (Fig. 4C). These results demonstrated that PLGA@M prefered to reside in inflammation sites when compared to free drugs and uncoated PLGA NPs, which implied that PLGA@M had a favorable potential in targeted therapy application.

### Therapeutic efficacy of PLGA@M in the coronavirus infected mice

To demonstrate the therapeutic potential of PLGA@M in coronavirus infection, severe coronavirus infected mouse model was established by infecting mice with a lethal dose of MHV. The infected mice were randomly divided into three groups (n = 8 in each group) to receive an i.v. injection of different formulation (saline, PLGA-LPV NPs and PLGA-LPV@M respectively) at a LPV dose of 10 mg/kg once 2 day. Tested mice in saline group lost weight rapidly and none of them survived longer than 5 days, whereas at the endpoint of observation, 10% mice survived in PLGA-LPV NPs treated group and an improvement survival to 60% in the PLGA-LPV@M treated group. Moreover, weights in survival mice were reversed to normal levels gradually (Fig. 5C, D).

**Fig. 6:**
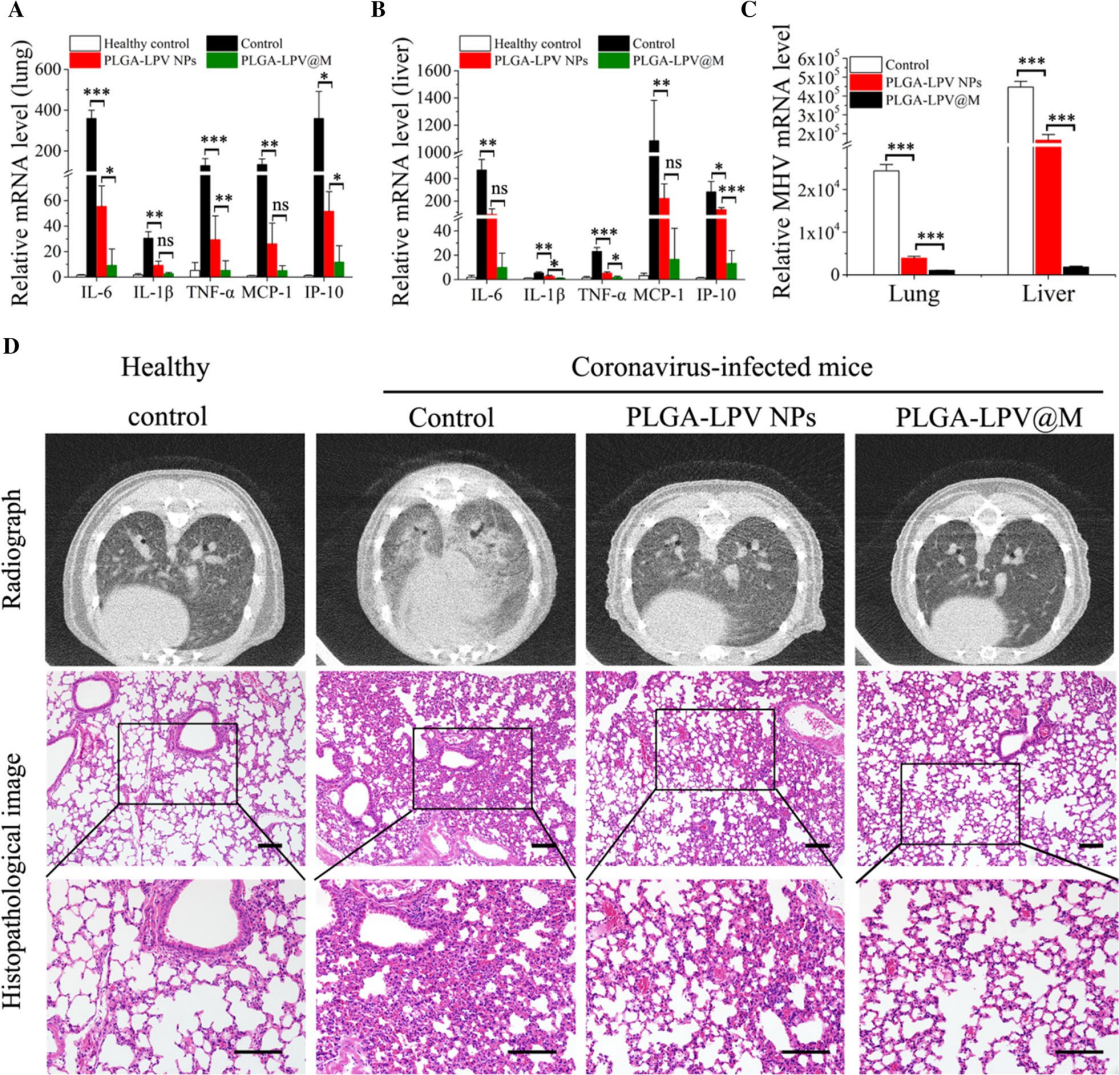
The efficacy of anti-inflammatory and antiviral by PLGA-LPV@M in vivo. The mRNA level of proinflammatory cytokines in the lung (**A**) and liver (**B**) tissues of coronavirus infectious mice with different treatment as measured by quantitative RT-PCR (n = 3). **C** The viral loads of lung and liver in coronavirus infectious mice after treated with PLGA-LPV NPs and PLGA-LPV@M (n = 3). **D** Histological and radiography analysis of lung derived from coronavirus infectious mice with different treatments. (Scale bar: 50 μm.) Data presented as mean ± s.d. *p ≤ 0.05, **p ≤ 0.01, ***p ≤ 0.001. *ns* not significant

In addition, therapeutic efficacy of all groups was further evaluated by investigating the inflammatory response and viral loading in main diseased organs in non-severe coronavirus infected mouse model. After 6 days treatment of PLGA-LPV NPs and PLGA-LPV@M, coronavirus infected mice were performed by CT scanner for radiography analysis. And then the treated mice were sacrificed to obtain lungs and livers for pathologic analysis, viral loads detection and inflammation measurement. Compared to the control and PLGA-LPV NPs treated groups, the lowest expression level of major proinflammatory cytokines (IL-6, IL-1β, TNF-α, MCP-1 and IP-10) in the lungs and the livers was observed in the PLGA-LPV@M treated group (Fig. 6A, B). Besides, we examined the viral loads by detecting the mRNA expression level of the virus in the tissues of lungs and livers. Indeed, PLGA-LPV@M reduced the viral loads significantly compared with PLGA-LPV NPs treated group (Fig. 6C), which was most likely owing to the targeted therapeutic efficacy of PLGA-LPV@M guided by membrane. Importantly, histological and radiography analysis also confirmed a less of inflammatory damage in the lungs and livers in PLGA-LPV@M treated group (Fig. 6D, Additional file 1: Fig. S6). These results together validated that PLGA-LPV@M could alleviate inflammatory response and reduce virus replication, thereby inhibited the inflammatory damage in organs and increased the survival rate of mice.

## Conclusion

In summary, we illustrated a treatment strategy for COVID-19 via macrophage biomimetic nanocarriers (PLGA@M) and their drug delivery system. PLGA@M inherited the membrane antigenic profile from macrophages and disguised as a mini macrophage to absorb multiple proinflammatory substances competitively. After that, blocked proinflammatory substances failed to activate immune cells, which alleviated the progression of CSS eventually. Furthermore, macrophage-like PLGA@M could carry drugs homing to the site of virus infection by the inflammatory milieu and the EPR effect, which promoted the local accumulation of drugs in infected tissues, thus enhance the effectiveness of pharmacotherapy, and reduce the adverse drug reaction. Owing to the synergistic effects of anti-inflammation and targeted antiviral treatment, LPV loaded PLGA@M exhibited significantly therapeutic effect in the mouse model of coronavirus infection, suggesting that PLGA@M may have similar treatment results when applied in COVID-19.

In severe COVID-19, excessive proinflammatory cytokines and continued infection resulted in uncontrolled formation of NETs, which induced the harmful amplification loop between inflammation and tissue damage [64, 65]. For these reasons, NETs were proposed as novel therapeutic targets in COVID-19 [66, 67]. In this study, PLGA@M manifested the talent in suppressing the formation of NETs induced by COVID-19 patient serum, suggesting that PLGA@M could play a multi-role in the treatment of COVID-19, and offer a comprehensive therapeutic benefit to patients. Meanwhile, NETs also engaged in the progression of multiple diseases including cardiovascular diseases [56, 68], rheumatoid arthritis [69], diabetes [70], cancer [71, 72] and so on, which meant that PLGA@M may have a potential to treat these diseases as well. However, the detailed mechanisms of how PLGA@M remove NETs and the possibility of clinical application still needed further exploration.

## Methods

### Cell culture

THP-1 cell lines (Human Monocyte Leukemia Cells) were purchased from the American Type Culture Collection (ATCC) and cultured in Roswell Park Memorial Institute (RPMI) Medium 1640 (Gibco, USA) supplemented with 10% fetal bovine serum (FBS, Hyclone) and 1% (v/v) penicillin/streptomycin (P/S, Gibco) at 37 °C in 5% CO_2_ environment. RAW 264.7 cell lines (Mouse Monocyte-macrophage Leukemia Cells) were purchased from the American Type Culture Collection (ATCC), and maintained in Dulbecco’s modified Eagle medium (DMEM) (Gibco) supplemented with 10% fetal bovine serum (FBS, Hyclone) and 1% (v/v) penicillin/streptomycin (P/S, Gibco) at 37 °C in 5% CO_2_ environment. L929 cell lines (Mouse Fibroblasts Cells) were purchased from the American Type Culture Collection (ATCC), and maintained in Dulbecco’s modified Eagle medium (DMEM) (Gibco) supplemented with 10% Heat-inactivated horse serum (Gibco) and 1% (v/v) penicillin/streptomycin (P/S, Gibco) at 37 °C in 5% CO_2_ environment. Human neutrophils were isolated from peripheral blood mononuclear cells (PBMCs) of healthy donors and cultured in serum-free RPMI 1640 at 37 °C in 5% CO_2_ environment.

### Cell membranes derivation

The plasma membranes were collected following a previously published method [24, 25]. Briefly, THP-1 or RAW 264.7 cells were cultured in T-175 culture flasks to full confluence, and the cells were collected by centrifugation at 700*g* for 5 min. The cells were washed with 1× PBS three times (500*g* for 10 min) and the cell pellets were then dispersed in homogenization buffer containing 75 mM sucrose, 20 mM Tris·HCl (pH = 7.5), 2 mM MgCl_2_ (Sigma, USA), 10 mM KCl (Sigma), and one tablet of protease/phosphatase inhibitors (Thermo Fisher, USA). The cell suspension was loaded into a dounce homogenizer and disrupted 15–25 passes. Following the disruption, the mixture was spun down at 800*g* for 5 min to collect the supernatant, and centrifuged again at 10,000*g* for 25 min to collect the supernatant again. Next, the collected supernatant was centrifuged at 150,000*g* for 50 min, and the plasma membrane pellets were collected after the supernatant was discarded, and then the plasma membrane pellets were washed once with water. Membrane protein contents were quantified with a Pierce BCA assay (Thermo Fisher). Then the membranes were stored in − 80 °C fridges for subsequent experiments.

### Preparation and characterization of PLGA@M

PLGA@M was synthesized in two steps. For the first step, PLGA cores were formulated using poly (d, l-lactide-*co*-glycolide) (50:50 PLGA, Aladdin, China) through a nanoprecipitation method. Briefly, 10 mg PLGA was first dissolved in 1 mL acetone, and then 3 mL of water was added rapidly. The solution was then placed in the fume hood and stirred for 4 h to remove the organic solvent. To load Lopinavir (LPV, Meilune, China) or Indocyanine green (ICG, Aladdin, China) into PLGA cores, 0.5 mg LPV or 0.6 mg ICG was mixed separately with 10 mg PLGA in 1 mL acetone. In the second step, PLGA cores were mixed with membranes at a polymer-to-membrane protein weight ratio of 1:0.5. The mixture was then interacted by using a bath sonicator with a frequency of 42 kHz and a power of 100 W for 5 min. After coated with membranes, PLGA@M was purified by centrifugation at 16,000*g* for 10 min to remove unbound membrane fragments. Then nanoparticles were measured for hydrodynamic size and surface zeta potential with Zetasizer (Malvern, UK). The stability of nanoparticles in 50% serum and 1× PBS was examined within 72 h. Nanoparticles were confirmed with transmission electron microscopy (TEM, JEM-1400 PLUS 120 kV). Briefly, 3 μL of nanoparticle suspension (1 mg/mL) was deposited onto a glow-discharged carbon-coated copper grid. Five minutes after the sample was deposited, the grid was rinsed with 10 drops of distilled water, and then stained with a drop of 1 wt% uranyl acetate. The grid was subsequently dried and visualized by TEM.

### Membrane protein characterization

The protein profile of cell lysate, membrane vesicles, PLGA NPs and PLGA@M were examined using sodium dodecyl sulfate polyacrylamide gel electrophoresis (SDS-PAGE). Specifically, samples were prepared at a protein concentration of 2.0 mg/mL in loading buffer (Fdbio, China), and separated by 10% SDS-PAGE, and then stained with coomassie brilliant blue.

For western blot analysis, all samples were mixed with loading buffer to the same total protein concentration of 2 mg/mL, and separated with 10% SDS-PAGE. Then the SDS-PAGE was transferred to a supported nitrocellulose membrane (Pall Life Sciences, Ann Arbor, MI, USA) and blocked with 5% BSA in PBS with 0.1% Tween 20 (PBST). Then, the blots were probed with specific antibodies for rabbit anti-human IL-6 receptor (Abcam, UK), rabbit anti-human IL-1β receptor (Abcam, UK) and rabbit anti-human ACE II (Abcam, UK). Corresponding horseradish peroxidase (HRP)-conjugated secondary antibodies were used to visualize by an enhanced chemiluminescent (ECL) reaction.

### Neutralizing cytokines by PLGA@M in vitro

Human and mouse recombinant IL-6 (1600 pg/mL for human IL-6, 1300 pg/mL for mouse IL-6, PeproTech, USA) and IL-1β (1700 pg/mL for human IL-1β, 4500 pg/mL for mouse IL-1β, PeproTech, USA) were mixed with PLGA@M derived from THP-1 or RAW 264.7 cells at different concentrations (0, 1, 2, 4 mg/mL). The mixtures were then incubated at 37 °C for 2 h. After the incubation, the mixtures were centrifuged at 21,000*g* for 15 min to remove the nanoparticles. Cytokine concentration in the supernatant was quantified with corresponding ELISA kits (DAKEWE, China). All experiments were carried out in triplicate.

### Inhibition of macrophage and neutrophil activation in vitro

THP-1 cells were seeded in 12-well culture plates at a density of 1 × 10^5^ cells per well. Human recombinant IL-6 (100 ng/mL) was incubated with PLGA@M (2 mg/mL) at 37 °C for 2 h. After the incubation, nanoparticles were removed by centrifuging at 21,000*g* for 15 min, and then the supernatant was added into THP-1 cell culture plates for another 2 h incubation. After that, the protein of p-STAT3 and STAT3 in THP-1 cells were analyzed by western blot (rabbit anti-human p-STAT3 and STAT3, Abcam, UK).

Neutrophils were seeded in 24-well plates at a density of 1 × 10^5^ cells/well. Human recombinant IL-1β (10 ng/mL) was incubated with PLGA@M (2 mg/mL) at 37 °C for 2 h. After the incubation, the mixtures were centrifuged at 21,000*g* for 15 min to remove the nanoparticles and the supernatant was added into neutrophils culture plates. The treated neutrophils were then fixed with 4% paraformaldehyde for 30 min at room temperature. After that, cells treated with 0.1% Triton X-100 in 1× PBS, and incubated with rabbit anti-human MPO (Abcam, UK) overnight. After washing three times with 1× PBS, the cells were incubated with secondary anti-rabbit antibody Zymosan Alexa Fluor 488 Fluorescent (Thermo Fisher, USA) for 2 h. Finally, the cell nucleus was stained by DAPI (Thermo Fisher, USA), and observed under inverted fluorescence microscope (Olympus DP80, Japan).

### Establishment of cellular model of SARS-CoV-2 pseudovirus infection

In order to obtain pseudotyped lentiviral particles expressing spike protein of SARS-CoV-2 (S-pseudovirus), pcDNA3.1-spike, pCDH-CMV-MCS-EF1-GFP-Puro vector and psPAX2 were co-transfected into 293 T cells. After 48 h, the lentiviral viruses were collected in the supernatant of the medium. S-pseudoviruses were concentrated by PEG8000 lentivirus concentrate, and the titer of pseudoviruses was detected by colloidal gold kit (Biodragon, China).

To establish the cellular model of S-pseudoviruses infection, human pulmonary epithelial cell lines (A549 cells) were infected with the S-pseudoviruses (MOI = 5), and the levels of inflammation were determined by quantifying the expression of main inflammatory cytokines in A549 by RT-PCR. The supernatant of S-pseudoviruses infected A549 cells (IS) was collected for subsequently experiment.

### Inhibition of the COVID-19 related proinflammatory factors in macrophage

THP-1 cells were seeded in 12-well culture plates containing 1 × 10^5^ cells per well. Inflammatory supernatant (IS) derived from S-pseudoviruses infected A549 was incubated with PLGA@M (2.0 mg/mL) at 37 °C for 2 h. After the incubation, the mixture was centrifuged at 21,000*g* for 15 min to remove the nanoparticles, and the supernatant was collected to activate THP-1 cells. Then the expression of inflammatory cytokines in THP-1 cells was evaluated using RT-PCR.

### Suppression of COVID-19 patient serum induced NETosis in neutrophils

Neutrophils were seeded in 24-well plates at a density of 1 × 10^5^ cells/well. COVID-19 patient serum was incubated with PLGA@M at different concentrations (0, 0.5, 1.0 mg/mL) at 37 ℃ for 1 h, and centrifuged for 15 min (17,000*g*) to collect the supernatant, and then the supernatant was added into neutrophils for 4 h. After that, NETosis in neutrophils were stained with Quant-iT™ PicoGreen™ dsDNA Assay Kit (Solarbio, China), which was then quantified with EnVision Multilabel Reader (PerkinElemer, UK) or observed under inverted fluorescence microscope (Olympus DP80, Japan).

### In vitro antiviral efficacy of PLGA-PLV@M

Mouse hepatitis virus (MHV, strain A59) was introduced to verify the antiviral activity of PLGA-LPV@M. Firstly, L929 cells were seeded in 12-well culture plates containing 1 × 10^5^ cells per well. Afterwards, MHV (MOI = 1) was mixed with PLGA-LPV NPs or PLGA-LPV@M at different LPV concentrations (0, 2.5, 5, 10, 20 μg/mL) for 1 h, and then the mixture was added into L929 cells, and the culture plates were shaken every 15 min to ensure the uniform distribution of MHV. After 2 h incubation, the culture supernatant was discarded and DMEM containing 2% FBS was added for further culture. After 24 h, cells and culture supernatant were collected respectively. The collected cells were performed by RT-PCR assay to detect the mRNA expression level of MHV, and the collected supernatant was used for plaque-forming units (PFU) assay to determine the titer of MHV.

For PFU assay, 1 mL culture supernatant (diluted 10^5^ times) was incubated with L929 cells for 2 h, and shook the culture plates every 15 min to ensure the uniform distribution of MHV. After 2 h, the culture supernatant was discarded and the cells were washed with 1× PBS for three times. Then a semi-solid medium composed of a 1:1 mixture of 2× DMEM and 2% Methyl cellulose supplemented with 4% FBS was added into the culture plates. After 2 days of incubation, the infected cells were fixed with 8% neutral buffered formalin (Solarbio, China) for 1 h, and then stained with a solution of 0.2% Gentian Violet (Solarbio, China), and enumerated the plaques.

### Targeting efficacy of PLGA@M in coronavirus infectious mouse model

To establish coronavirus infected mouse mode, 6-week-old BALB/c female mice (purchased from Guangdong Medical Experimental Animal Center) were anesthetized with 4% chloral hydrate intraperitoneally (Sigma), and then inoculated intranasal with 15 μL of MHV (2.5 × 10^5^ PFU). Two days after intranasal inoculation, infected mice were sacrificed, and the main organs were collected for pathologic analysis.

To evaluate targeting efficacy of PLGA@M, indocyanine Green (ICG, Sigma) loaded PLGA NPs, ICG loaded PLGA@M and free ICG (200 μL, 100 ug/mL) were injected intravenously into the coronavirus infected mice. After 12 h, treated mice were sacrificed, lungs and livers were collected and imaged with the IVIS LuminaIII Series system (PerkinElmer).

### Therapeutic efficacy of PLGA-LPV@M in coronavirus infectious mice

To demonstrate the therapeutic potential of PLGA@M in coronavirus infectious mice, severe coronavirus infected mouse model was established by infecting mice with a lethal dose of MHV (5 × 10^5^ PFU) with a method of nasal drip. Two days after infection, infected mice were treated with 200 μL 1× PBS, PLGA-LPV NPs (100 μg/mL for LPV concentration) and PLGA-LPV@M (100 μg/mL for LPV concentration) every day, and the weight and mortality of mice were recorded every day until the end of experimental point.

To explore the anti-inflammatory and antiviral effect of PLGA-LPV@M in vivo, coronavirus infected mice were treated with 200 μL 1× PBS, PLGA-LPV NPs (100 μg/mL for LPV concentration) and PLGA-LPV@M (100 μg/mL for LPV concentration) once 2 days. After 6 days post treatments, all group mice were performed with radiography analysis by CT (nanoScan PET/CT, MEDISO, Hungary). Then the mice in each group were sacrificed, and the lungs and livers were collected and rinsed in ice-cold 1× PBS. Part of the tissue was taken for H&E staining for pathologic analysis, the other part was homogenized with zirconia beads in 1 mL 1× PBS using the Tissue Lyser II instrument (QIAGEN, GER), and the mRNA expression of MHV and inflammatory factors was detected by RT-PCR assay.

### RT-PCR

Total RNA was isolated with Trizol reagent (Invitrogen, USA) according to the manufacturer’s instruction, the quantitative RT-PCR reaction was performed in a MyiQ cycler (Bio-Rad, USA) using SYBR Green I (Molecular Probes, USA). The PCR primers were purchased from Beijing Genomics Institute, and the sequence of primers were shown in Additional file 1: Table S1. The mRNA levels of indicated genes were normalized to that of β-Actin mRNA.

### Safety study

To investigate cellular biocompatibility of PLGA@M, THP-1 cells and RAW 264.7 cells were placed in 96 well plates with 5 × 10^3^ cells/well respectively. PLGA@M was added into cell plates at different concentrations (0, 0.125, 0.25, 0.5, 1, 2, 4 mg/mL) for 24 h. After that, cell viability was evaluated by CCK8 kits (Meilunbio, China), and the optical density was measured at 450 nm with a microplate spectrophotometer (Biotek, USA).

To evaluate the safety of PLGA@M in vivo, BALB/c female mice were i.v. injected through the tail vein with 200 μL of PLGA@M (4 mg/mL) or saline once every 2 days for 1 week. After 21 days treatment, serum was collected from sacrificial mice for liver and renal function indices and inflammatory cytokine investigation. In addition, heart, liver, spleen, lung and kidney were collected from the same mice, and fixed in 10% formalin for 24 h, and sectioned for hematoxylin and eosin (H&E) staining.

### Statistical analysis

All experiments were carried out in triplicate, and the results are expressed as the mean ± standard deviation of the mean value (SD). Unless otherwise indicated, statistical analysis was performed with Student’s unpaired t-test using GraphPad Prism 6.0 (USA). The differences were considered to be statistically significant at *p* < 0.05. Kaplan–Meier survival analyses were performed to analyze the survival rate.

## Supplementary Information

**Additional file 1: Fig. S1.** Hydrodynamic diameter measurements of PLGA@M in water and in 1× PBS. PLGA@M prepared with various polymer-to-membrane protein weight ratios. **Fig. S2. **Hydrodynamic diameter measurements of PLGA NPs, PLGA-LPV NPs, and PLGA-LPV@M by dynamic light scattering. **Fig. S3. **The mean fluorescence intensity of MPO in neutrophils with different treatments, data was quantified by image J. **Fig. S4. **Cell viability evaluation of RAW264.7 after incubation with the mice macrophage derived membrane PLGA@M in different concentrations. **Fig. S5.** Binding capacity of PLGA@M with mice recombinant IL-6 and IL-1β. **Fig. S6. **Histological analysis of livers derived from coronavirus infectious mice with different treatments. **Table S1.** Primers sequences used in the RT-PCR experiments in this work.

## Data Availability

The datasets used and/or analyzed during the current study are available from the corresponding author on reasonable request.

## Abbreviations

COVID-19: Coronavirus disease 2019
SARS-CoV-2: Severe acute respiratory syndrome coronavirus 2
CSS: Cytokine storm syndrome
NETs: Neutrophil extracellular traps
ACE II: Angiotensin-converting enzyme 2
LPV: Lopinavir
EPR effect: Enhanced permeability and retention effect
IL-6R: IL-6 receptor
IL-1R: IL-1 receptor
ELISA: Enzyme-linked immunosorbent assay
MHV: Mouse hepatitis virus
ALT: Alanine aminotransferase
AST: Aspartate aminotransferase
CRE: Creatinine
BUN: Blood urea nitrogen
ATCC: American Type Culture Collection
RPMI: Roswell Park Memorial Institute
DMEM: Dulbecco’s modified Eagle medium
ICG: Indocyanine green
