# Supplementary material for: Macrophage biomimetic nanocarriers for anti-inflammation and targeted antiviral treatment in COVID-19
Source: J Nanobiotechnology. 2021 Jun 10;19:173. doi: 10.1186/s12951-021-00926-0 (PMC8190731; doi:10.1186/s12951-021-00926-0)
Supplement: Supplementary file 1 — Additional file 1: Fig. S1. Hydrodynamic diameter measurements of PLGA@M in water and in 1× PBS. PLGA@M prepared with various polymer-to-membrane protein weight ratios. Fig. S2. Hydrodynamic diameter measurements of PLGA NPs, PLGA-LPV NPs, and PLGA-LPV@M by dynamic light scattering. Fig. S3. The mean fluorescence intensity of MPO in neutrophils with different treatments, data was quantified by image J. Fig. S4. Cell viability evaluation of RAW264.7 after incubation with the mice macrophage derived membrane PLGA@M in different concentrations. Fig. S5. Binding capacity of PLGA@M with mice recombinant IL-6 and IL-1β. Fig. S6. Histological analysis of livers derived from coronavirus infectious mice with different treatments. Table S1. Primers sequences used in the RT-PCR experiments in this work. [file 12951_2021_926_MOESM1_ESM.docx]

**Additional data for**

**Macrophage Biomimetic Nanocarriers for Anti-inflammation and Targeted Antiviral Treatment in COVID-19**

Qingqin Tan^1, 2, 3, †^, Lingjie He^1, 2, 3, †^, Xiaojun Meng^4^, Wei Wang^1^, Hudan Pan^5^, Weiguo Yin^6^, Tianchuan Zhu^1^, Xi Huang^1, 2, 3, 6,^ * and Hong Shan ^1,^ *

^1^ Center for Infection and Immunity, Guangdong Provincial Key Laboratory of Biomedical Imaging, The Fifth Affiliated Hospital of Sun Yat-sen University, Zhuhai 519000, Guangdong, China.

^2^ Southern Marine Science and Engineering Guangdong Laboratory, Zhuhai 519000, Guangdong, China.

^3^ Key Laboratory of Tropical Diseases Control, Ministry of Education, Zhongshan School of Medicine, Sun Yat-sen University, Guangzhou 510080, Guangdong, China.

^4^ Department of Endocrinology, The Fifth Affiliated Hospital of Sun Yat-sen University, Zhuhai 519000, Guangdong, China.

^5^ Dr. Neher's Biophysics Laboratory for Innovative Drug Discovery, State Key Laboratory of Quality Research in Chinese Medicine, Macau University of Science and Technology, Macao 999078, China.

^6^ The Sixth Affiliated Hospital of Guangzhou Medical University, Qingyuan People's Hospital, Qingyuan 511518, Guangdong, China.

* Correspondence author: huangxi6@mail.sysu.edu.cn (Xi Huang), shanhong@mail.sysu.edu.cn (Hong Shan)

^†^ These authors contributed equally to this work


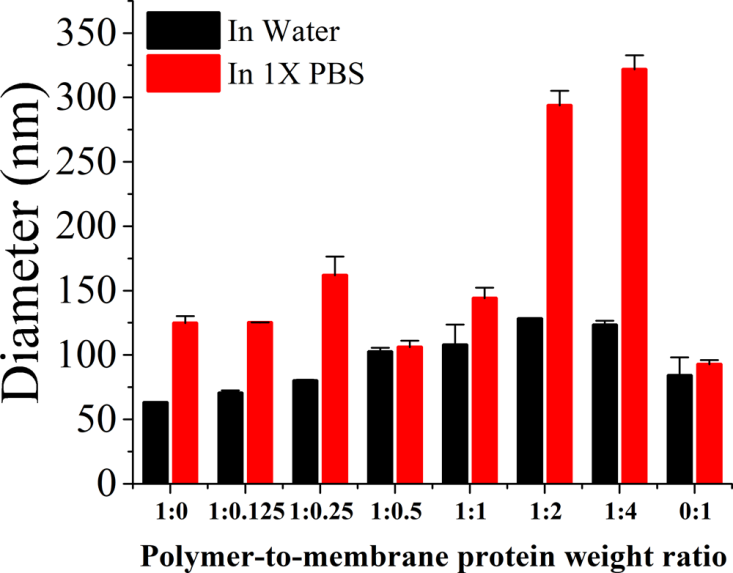


**Fig. S1.** Hydrodynamic diameter measurements of PLGA@M in water and in 1xPBS. PLGA@M prepared with various polymer-to-membrane protein weight ratios.


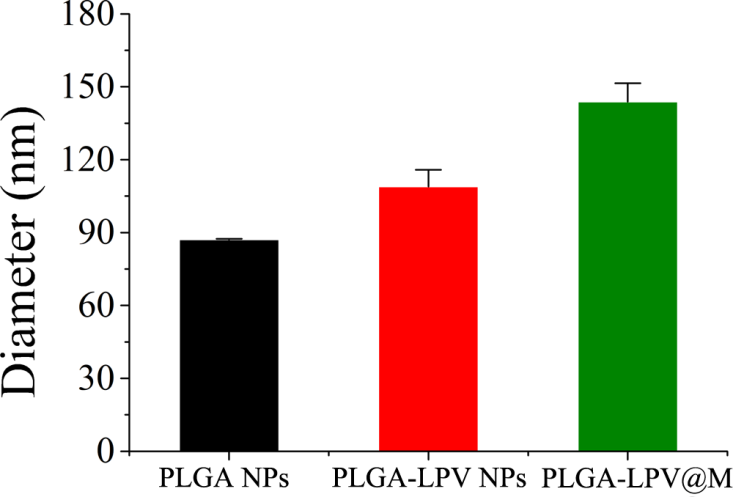


**Fig. S2.** Hydrodynamic diameter measurements of PLGA NPs, PLGA-LPV NPs, and PLGA-LPV@M by dynamic light scattering.


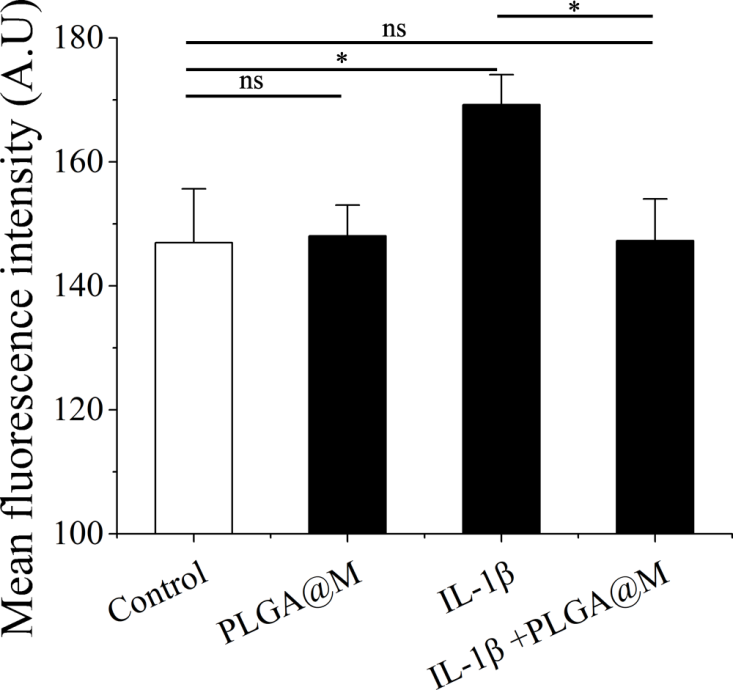


**Fig. S3.** The mean fluorescence intensity of MPO in neutrophils with different treatments, data was quantified by image J (n=3)**.** Data presented as mean ± s.d. **p* ≤ 0.05, ***p* ≤ 0.01, ****p* ≤ 0.001. ns, not significant.


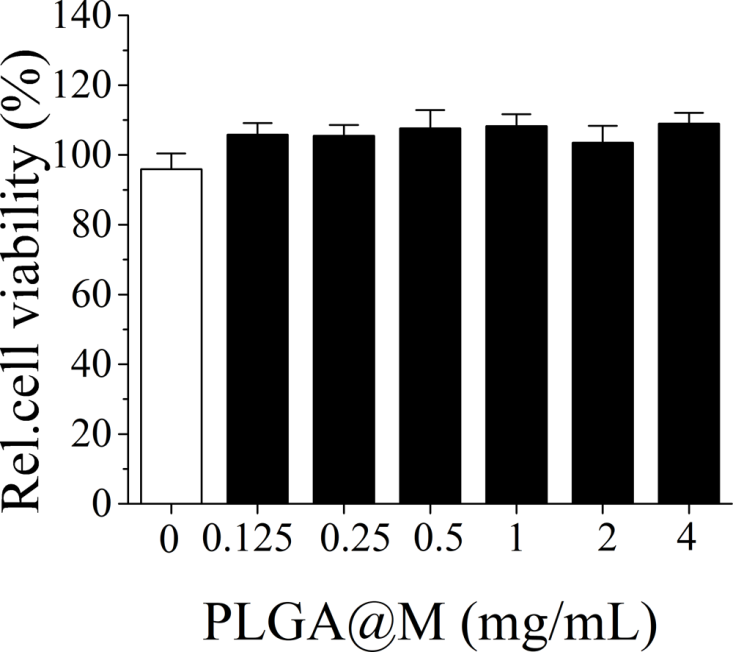


**Fig. S4.** Cell viability evaluation of RAW264.7 after incubation with the mice macrophage derived membrane PLGA@M in different concentrations (n=6).


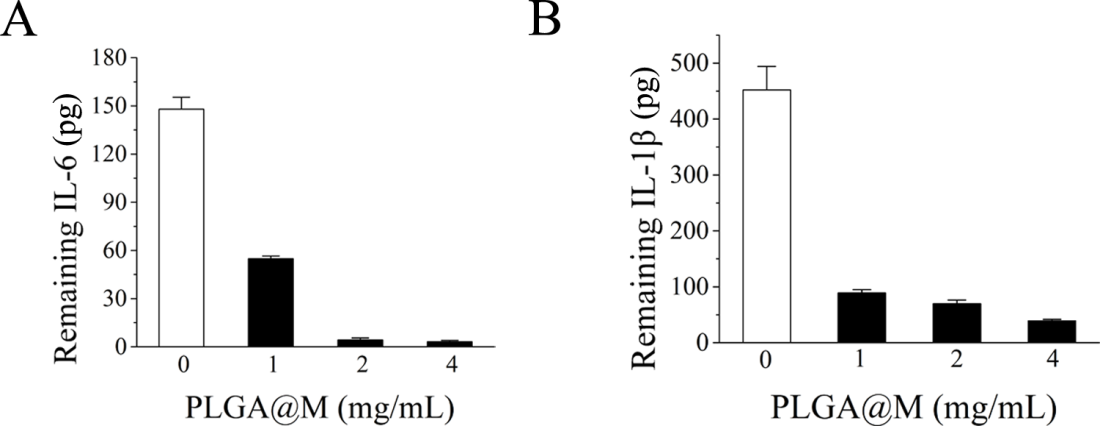


**Fig. S5.** Binding capacity of PLGA@M with mice recombinant (E) IL-6 and (F) IL-1β (n=3).

**
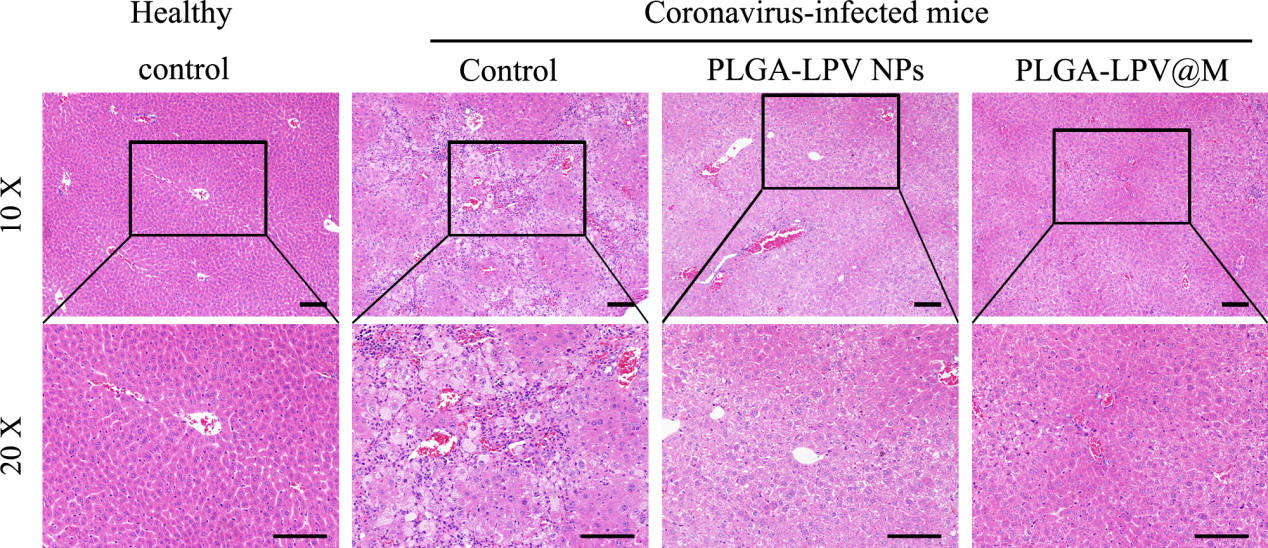
**

**Fig. S6.** Histological analysis of livers derived from coronavirus infectious mice with different treatments. (Scale bar: 50 μm.)

**Table S1.** Primers sequences used in the RT-PCR experiments in this work.

| **Primer** | **Sequence (5’-3’)** |
| --- | --- |
| Human IL-6-5F | AGACAGCCACTCACCTCTTCAG |
| Human IL-6-3R | TTCTGCCAGTGCCTCTTTGCTG |
| Human IL-1β-5F | CCACAGACCTTCCAGGAGAATG |
| Human IL-1β-3R | GTGCAGTTCAGTGATCGTACAGG |
| Human TNF-α-5F | CTCTTCTGCCTGCTGCACTTTG |
| Human TNF-α-3R | ATGGGCTACAGGCTTGTCACTC |
| Human β-Actin-5F | GCTCCTCCTGAGCGCAAG |
| Human β-Actin-3R | CATCTGCTGGAAGGTGGACA |
| MHV-A59-5F | CGG AAT TCG GGT TGA TGT CTT GTG TAC TG |
| MHV-A59-3R | CCG CTC GAG TTA CAA TTT AAA GTT GGT ATAGAC |
| Mouse IL-6-5F | CACAGAGGATACCACATCCCAACA |
| Mouse IL-6-3R | TCCACGATTTCCCAGAGAACA |
| Mouse IL-1β-5F | CAACCAACAAGTGA TATTCTCCATG |
| Mouse -IL-1β-3R | GATCCACACTCTCCAGCTGCA |
| Mouse TNF-α-5F | GGTGCCTATGTCTCAGCCTCTT |
| Mouse TNF-α-3R | CGATCA CCCCGAAGTTCAGTA |
| Mouse MCP-1-5F | TGGGTCCAGACATACATT |
| Mouse MCP-1-3R | ACGGGTCAACTTCACATT |
| Mouse IP-10-5F | GCTGGGTCTGAGTGGGA |
| Mouse IP-10-3R | ACGTGGGCA GGATAGGC |
| Mouse β-Actin-5F | GATTACTGCTCTGGCT CCTAGC |
| Mouse β-Actin-3R | GACTCATCGTACTCCTGCTTGC |
